# Neonatal Interfacility Transport to Tertiary and Quaternary Centres: Clinical Outcomes and System-Level Determinants—A Systematic Review

**DOI:** 10.3390/children13081064

**Published:** 2026-08-10

**Authors:** Roni Octavian Damian, Lidia Boldeanu, Mihai Gabriel Cucu, Mirela Anişoara Siminel, Mihai Alexandru Butoi, Vlad Ionuț Belghiru, Silvia Nica, Vlad Pădureanu, Mohamed-Zakaria Assani, Virginia Maria Rădulescu, Cristiana Geormaneanu, Mihail Virgil Boldeanu, Luciana Teodora Rotaru

**Affiliations:** 1 Doctoral School, University of Medicine and Pharmacy of Craiova, 200349 Craiova, Romania; roni.damian@umfcv.ro (R.O.D.); mihai.butoi@umfcv.ro (M.A.B.); vlad.belghiru@umfcv.ro (V.I.B.); mohamed.assani@umfcv.ro (M.-Z.A.); 2 Department of Emergency Medicine and First Aid, University of Medicine and Pharmacy of Craiova, 200349 Craiova, Romania; cristiana.geormaneanu@umfcv.ro (C.G.); luciana.rotaru@umfcv.ro (L.T.R.); 3 Department of Microbiology, Faculty of Medicine, University of Medicine and Pharmacy of Craiova, 200349 Craiova, Romania; lidia.boldeanu@umfcv.ro; 4 Laboratory of Human Genomics, University of Medicine and Pharmacy of Craiova, 200638 Craiova, Romania; 5 Department of Neonatology, University of Medicine and Pharmacy of Craiova, 200349 Craiova, Romania; 6 Department of Emergency and First Aid, Faculty of Medicine, Carol Davila University of Medicine and Pharmacy, 020021 Bucharest, Romania; silvia.nica@umfcd.ro; 7 University Emergency Hospital of Bucharest, 050098 Bucharest, Romania; 8 Department of Internal Medicine, Faculty of Medicine, University of Medicine and Pharmacy of Craiova, 200349 Craiova, Romania; vlad.padureanu@umfcv.ro; 9 Department of Immunology, Faculty of Medicine, University of Medicine and Pharmacy of Craiova, 200349 Craiova, Romania; mihail.boldeanu@umfcv.ro; 10 Department of Medical Informatics and Biostatistics, Faculty of Medicine, University of Medicine and Pharmacy of Craiova, 200349 Craiova, Romania; virginia.radulescu@umfcv.ro

**Keywords:** neonatal transport, outborn infants, tertiary neonatal care, quaternary neonatal care, neonatal mortality, neonatal morbidity, premature infants, interfacility transfer, hypothermia, TRIPS score, transport-associated adverse events, regionalised perinatal care, neurodevelopmental outcomes

## Abstract

**Highlights:**

**What are the main findings?**

- Outborn neonatal transfer was generally associated with greater mortality and major morbidity, particularly among very preterm infants, although the magnitude varied across care systems.
- Hypothermia, cardiorespiratory instability, and haemodynamic deterioration were recurrent and potentially modifiable transport-related complications.

**What are the implications of the main findings?**

- Specialised transport teams, structured stabilisation protocols, and thermal-management bundles may improve physiological stability during transfer.
- Harmonised outcome definitions and prospective multicentre studies are needed to support benchmarking and strengthen causal inference.

**Abstract:**

**Background/Objectives**: Outborn neonates—those born outside tertiary perinatal centres and transferred postnatally—may experience higher mortality and morbidity than inborn infants. This systematic review without meta-analysis synthesised contemporary evidence on neonatal interfacility transfer to tertiary and quaternary neonatal centres, focusing on mortality, major morbidity, physiological instability, prognostic tools, and organisational determinants. **Methods**: Four electronic databases (PubMed/MEDLINE, Scopus, Web of Science, and the Cochrane Library) were searched for English-language publications from January 2010 to December 2025. Study selection and data extraction were performed independently by two reviewers, with disagreements resolved by consensus. Narrative synthesis followed PRISMA 2020 and SWiM principles. Of the 2456 records identified, 44 publications were retained: 31 primary studies and 13 contextual or methodological sources. **Results**: Across the primary evidence, outborn status was generally associated with higher mortality and major morbidity, particularly among very preterm infants, although effect magnitude varied across healthcare systems. Hypothermia, respiratory deterioration, and haemodynamic instability were recurrent transport-related complications. Specialised teams, standardised stabilisation, and thermal-management bundles were associated with better physiological stability. TRIPS and TRIPS-II showed prognostic utility. **Conclusions**: Predominantly observational evidence suggests that neonatal transport outcomes reflect interactions between biological vulnerability, transport-related stress, and system organisation. Strengthening regionalised transport pathways and standardising stabilisation practices may improve outcomes, but causal inference remains limited.

## 1. Introduction

Neonatal service designations are not uniform internationally. Under the American Academy of Pediatrics framework, Level I facilities provide routine newborn care; Level II units care for moderately ill or premature infants who do not require prolonged intensive support; Level III units provide sustained life support and comprehensive care for critically ill and very preterm neonates; and Level IV centres meet Level III capabilities while also providing on-site management of complex medical and surgical conditions [1]. Other health systems use functional designations—for example, special care units, local neonatal units, and neonatal intensive care units in the United Kingdom—rather than a universal four-level scheme. Accordingly, this review uses the terms tertiary and quaternary neonatal centres functionally and refers to Level III or IV only when this designation was used by the original study [1,2].

Despite these advances, a substantial proportion of preterm and critically ill neonates continue to be delivered outside tertiary centres and subsequently require postnatal transfer. This is particularly evident in healthcare systems with incomplete regionalisation, geographic barriers, or limited resource availability [3,4,5]. As a result, neonatal transport has become an essential component of perinatal care systems worldwide.

Outborn neonates represent a particularly vulnerable population. Cohort studies have reported higher mortality among very preterm infants transferred after birth than among inborn infants, including after adjustment for gestational age and illness severity [6,7,8,9,10]. Higher rates of severe intraventricular haemorrhage, necrotising enterocolitis, bronchopulmonary dysplasia, and sepsis have also been reported, although findings vary across settings [7,9,10]. Prospective referred-neonate cohorts from India and Ethiopia further identified hypothermia, hypoglycaemia, impaired perfusion or oxygenation, low admission weight, and prolonged travel as predictors of mortality [11,12].

These differences cannot be attributed solely to birth location. Growing evidence suggests that neonatal transport itself constitutes a distinct high-risk clinical phase in which physiological instability is common and clinically consequential. Adverse events during transfer include hypothermia, respiratory deterioration, hypoglycaemia, and haemodynamic instability, all of which may contribute to unfavourable short- and long-term outcomes [3,13,14].

Among these complications, hypothermia has emerged as one of the most consistent and clinically impactful transport-related risks. Studies have reported high rates of admission hypothermia following neonatal transfer, particularly in settings without standardised thermal protection protocols [13,15]. Hypothermia remains one of the most frequently reported complications during neonatal transport and has been consistently associated with increased morbidity and mortality. Effective thermal management is therefore considered a key component of neonatal transport quality and stabilisation. Transport-related factors, such as duration, equipment availability, and team expertise, significantly influence thermal stability and overall physiological status upon arrival [16,17].

To better quantify transport-related risk, physiological scoring systems such as the Transport Risk Index of Physiologic Stability (TRIPS) and its updated version (TRIPS-II) have been developed and validated [18,19]. Higher scores at admission, and particularly worsening physiological status during transport, have been consistently associated with increased early neonatal mortality and adverse neurodevelopmental outcomes at 18–24 months of corrected age [20]. Comparative analyses of multiple transport scoring systems have further confirmed the validity of physiology-based scores for risk stratification in outborn neonates [21].

Beyond individual clinical parameters, system-level factors—including regionalisation of care, availability of specialised transport teams, protocol standardisation, and interfacility communication—play a crucial role in determining outcomes. Variability in these factors across healthcare systems contributes to the heterogeneity observed in the literature and limits the generalisability of individual findings [14,22,23,24]. Evidence from well-organised regional transport services further demonstrates that adverse outcomes are substantially modified by team composition, equipment, and coordinated care pathways [25,26,27].

Given the complexity of these interactions and the variability of existing evidence, a structured synthesis is needed to clarify the relative contribution of birth setting, transport-related factors, and system-level determinants to neonatal outcomes.

The aim of this review was to evaluate associations between postnatal neonatal transfer to tertiary or quaternary centres and mortality, morbidity, and other clinical outcomes, and to examine transport-related physiological instability, severity-assessment tools, and system-level determinants of neonatal transport.

## 2. Materials and Methods

### 2.1. Review Design and Conceptual Framework

This study was designed as a systematic review without meta-analysis to synthesise contemporary evidence on neonatal interfacility transfer to tertiary or quaternary neonatal centres. The review focused on mortality, major neonatal morbidity, transport-related physiological instability, prognostic assessment tools, and organisational determinants of transport safety and outcomes.

A structured narrative synthesis approach was selected because the available literature was characterised by substantial clinical, methodological, and organisational heterogeneity. Formal quantitative meta-analysis was not performed because the included studies differed considerably with respect to:

- Study populations and gestational-age distributions;
- Definitions of outborn status;
- Transport systems and referral structures;
- Transport modalities and stabilisation practices;
- Outcome definitions;
- Analytical methodologies.

Given this heterogeneity, together with the predominance of observational study designs, formal quantitative meta-analysis was considered methodologically inappropriate and potentially misleading. Instead, the review aimed to identify consistent patterns across the literature, integrate evidence from diverse healthcare settings, and develop a clinically and organisationally meaningful synthesis of current knowledge.

The review was conducted and reported in accordance with the Preferred Reporting Items for Systematic Reviews and Meta-Analyses (PRISMA) 2020 statement [28]. Because quantitative pooling was not undertaken, the narrative synthesis was structured using the Synthesis Without Meta-analysis (SWiM) reporting principles [29], including transparent grouping of studies, explicit consideration of heterogeneity, and cautious interpretation of effect direction and magnitude.

The review protocol was not prospectively registered in PROSPERO, OSF, or another public registry. This departure from current best practice is reported transparently and considered when interpreting the methodological limitations of the review.

### 2.2. Eligibility Criteria

Eligibility criteria were predefined according to the objectives of the review and structured using a PICO framework: Population—neonates (birth to 28 days of life) requiring interfacility transfer; Intervention/Exposure—postnatal interhospital transfer to a tertiary or quaternary neonatal centre (AAP Level III/IV or locally equivalent); Comparator—inborn neonates managed at a tertiary centre without postnatal transfer, when available; Outcomes—neonatal mortality, major morbidity, transport-related physiological instability, illness severity, and system-level transport-quality indicators.

*Population*

Studies involving neonates (birth to 28 days of life) requiring interfacility transfer were considered eligible. Particular attention was given to:

- Preterm infants (<37 weeks’ gestation);
- Very preterm infants (<32 weeks);
- Very-low-birth-weight (VLBW) neonates (<1500 g);
- Extremely low-birth-weight (ELBW) neonates (<1000 g).

As these populations are most vulnerable to transport-related instability and adverse outcomes.

*Exposure of Interest*

The primary exposure of interest was postnatal interhospital transfer from a lower-capability facility to a tertiary or quaternary neonatal centre, irrespective of the local naming convention. Studies focusing exclusively on antenatal or in utero transfer were excluded unless postnatal transport data were reported separately.

*Outcomes*

The primary outcomes of interest included:

- Neonatal mortality;
- In-hospital mortality;
- Major neonatal morbidity, including severe intraventricular haemorrhage (IVH grade III–IV), necrotising enterocolitis (NEC ≥ Bell stage II), bronchopulmonary dysplasia (BPD), severe retinopathy of prematurity (ROP), periventricular leukomalacia (PVL), and culture-proven sepsis.

Secondary outcomes included:

- Transport-related physiologic instability;
- Hypothermia;
- Respiratory and haemodynamic deterioration;
- Transport-associated adverse events;
- Illness severity assessment using TRIPS or TRIPS-II;
- Transport process indicators;
- Long-term neurodevelopmental outcomes when available.

*Study Designs*

Eligible primary evidence comprised:

- Observational cohort studies;
- Case–control studies;
- Cross-sectional studies;
- Quality-improvement studies;
- Transport-system evaluations;
- Systematic reviews retained only for contextualisation and reference checking, without inclusion in the primary-study synthesis.

Editorials, narrative opinion pieces, conference abstracts without full data, isolated case reports, and case series including fewer than 10 patients were excluded.

Only studies published in English between January 2010 and December 2025 were included to ensure contemporary relevance to current neonatal transport practice and regionalised care systems.

### 2.3. Information Sources and Search Strategy

A comprehensive literature search was conducted in MEDLINE (via PubMed), Web of Science Core Collection, Scopus, and the Cochrane Library. Reference lists of relevant reviews and key primary studies were also screened to identify additional eligible publications.

Search concepts covered the neonatal population, interfacility transport or retrieval, tertiary neonatal care, and clinical or system-level outcomes. Controlled vocabulary and free-text terms included neonate, newborn, transport, transfer, retrieval, neonatal retrieval, transport service, transport team, interhospital transport, interfacility transport, and aeromedical transport.

(“Infant, Newborn”[MeSH] OR neonate* OR newborn*) AND (“Transportation of Patients”[MeSH] OR transport* OR transfer* OR retrieval OR “neonatal retrieval” OR “transport service” OR “transport team” OR “interhospital transport” OR “interfacility transport” OR “aeromedical transport”) AND (“Intensive Care Units, Neonatal”[MeSH] OR “level III” OR “level IV” OR tertiary OR quaternary OR “regionalized perinatal care”) AND (mortality OR morbidity OR outcome* OR “treatment outcome”[MeSH]) AND (2010/01/01:2025/12/31[dp])

The syntax and controlled vocabulary were adapted for each database. The initial search was conducted in September 2025 and updated in December 2025. The documented strategies were rerun in July 2026 to verify reproducibility and consolidate the record set; eligibility remained restricted to publications available through December 2025. Complete database-specific strategies, limits, platforms, and audit counts are provided in Supplementary Table S1.

### 2.4. Study Selection

Titles and abstracts were screened independently by two reviewers. Records judged potentially eligible or insufficiently described were assessed in full text by the same reviewers. Disagreements at either stage were resolved through discussion and consensus; no third reviewer was required.

The reproducibility audit identified 2456 database records. After removal of 1085 duplicate records, 1371 unique records underwent independent title-and-abstract screening. The contemporaneous review summary recorded 327 publications assessed in full text. After application of the eligibility criteria, 44 publications were retained: 31 primary studies contributed to the evidence synthesis, while 13 secondary or methodological sources were used only for contextualisation, reporting guidance, or reference checking.

The study selection process is illustrated in the PRISMA 2020 flow diagram.

### 2.5. Data Extraction and Management

Data were extracted independently by two reviewers using a standardised form. Discrepancies were reconciled through discussion and consensus. For each included primary study, the following data were extracted:

- Study characteristics (author, year, country, design, and study period);
- Population characteristics (gestational age and birth weight categories);
- Exposure details (inborn vs. outborn status, transfer indication, timing, referring and receiving facility level);
- Transport characteristics (mode, team composition, equipment, duration, and stabilisation procedures);
- Physiological status and illness severity (including TRIPS/TRIPS-II where available);
- Outcomes (mortality, morbidity, transport-related adverse events, length of stay, and long-term outcomes);
- Where possible, data were extracted separately for key subgroups (e.g., <28 weeks vs. 28–31 weeks; VLBW vs. ELBW; inborn vs. outborn; ground vs. air transport).

When overlapping cohorts were identified, the most recent or most comprehensive study was retained.

### 2.6. Risk of Bias and Quality Assessment

Given the predominance of observational studies, risk of bias was assessed using the Newcastle–Ottawa Scale for cohort and case–control studies [30]. Assessment focused on cohort selection, group comparability (particularly inborn versus outborn), and outcome ascertainment.

Key methodological aspects—including adjustment for confounders (gestational age, birth weight, illness severity), handling of missing data, and clarity of outcome definitions—were documented to support the interpretation of findings.

### 2.7. Data Synthesis

A quantitative meta-analysis was not performed because study designs, populations, transport systems, exposure definitions, outcome definitions, and analytical approaches were not sufficiently comparable.

A structured narrative synthesis was therefore conducted in accordance with SWiM principles and organised around the following prespecified clinical and system-level domains:

- Mortality and major morbidity differences between outborn and inborn infants;
- Transport-related physiological instability and adverse events;
- The role of specialised transport teams, stabilisation practices, and monitoring tools (including TRIPS/TRIPS-II);
- System-level factors and performance indicators of neonatal transport services.

Within each domain, primary studies were grouped by population, transport setting, and outcome construct. Effect estimates were reported as presented in the original publications, with emphasis on their direction, range, adjustment strategy, and clinical context rather than statistical significance alone. Conclusions were weighted qualitatively according to study design, sample size, confounder adjustment, risk of bias, consistency, and directness. Secondary reviews informed context and reference checking but were not combined with primary-study estimates, thereby avoiding double counting.

### 2.8. Assessment of Reporting Bias and Certainty of Evidence

Publication and selective-reporting bias were considered qualitatively by examining study design, sample size, outcome availability, direction of findings, and the predominance of positive quality-improvement reports. Funnel plots and statistical tests for small-study effects were not undertaken because no sufficiently homogeneous set of pooled estimates was available. Certainty was appraised at the outcome-domain level using study limitations, consistency, directness, precision, and risk of bias; a formal GRADE rating was not assigned because of marked heterogeneity and the absence of meta-analytic effect estimates.

## 3. Results

### 3.1. Study Selection and General Characteristics

**Figure 1:**
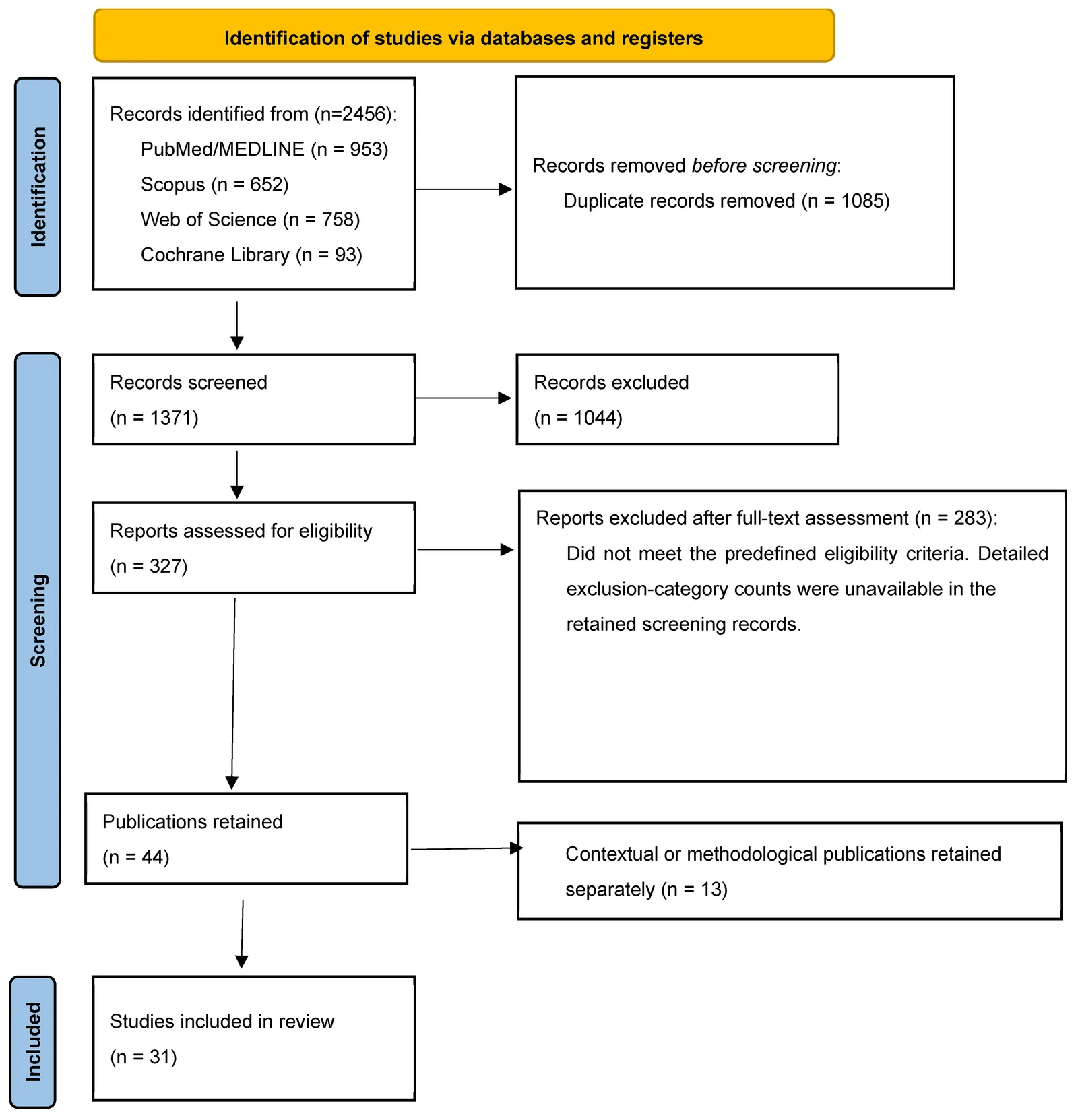
PRISMA 2020 flow diagram of study selection. Database-level identification and deduplication counts derive from the reproducibility audit; full-text-stage counts were reconstructed from the contemporaneous manuscript summary because the record-level screening log was unavailable. Accordingly, unsupported exclusion-category counts were removed and exclusions are reported only as a verified total. The 44 retained publications comprised 31 primary studies and 13 contextual or methodological sources; secondary sources were not counted as primary evidence.

A total of 2456 records were identified through database searching. After removal of 1085 duplicate records, 1371 records were screened by title and abstract, of which 1044 were excluded. Full-text assessment was performed for 327 publications; 283 were excluded after full-text review. Forty-four publications were retained: 31 primary studies contributed to the qualitative evidence synthesis and 13 contextual or methodological sources supported interpretation, reporting, and reference checking. The selection process is summarised in Figure 1.

The 44 retained publications covered five overlapping thematic domains: outborn versus inborn outcomes and regionalisation; transport systems, safety, and organisation; transport complications and stabilisation; hypothermia and thermal management; and transport-related severity or prognostic scoring. Table 1 describes the distribution of retained publications by their principal thematic contribution; these domain counts should not be interpreted as independent pooled evidence sets.

The primary evidence base comprised 31 empirical studies, predominantly observational cohorts, together with quality-improvement and transport-system evaluations. Thirteen secondary or methodological publications—including systematic reviews, reporting guidance, and consensus work—were retained separately for contextual interpretation and reference checking. Evidence originated from highly regionalised systems and resource-limited settings, although high-income countries predominated.

The principal outcomes assessed across the primary studies included mortality, major neonatal morbidity, physiological instability, hypothermia, transport-related adverse events, and the prognostic performance of severity scores. Sources of heterogeneity and differences in key outcome definitions are summarised in Supplementary Table S2.

Among cohort and case–control studies assessed with the Newcastle–Ottawa Scale, scores were generally 7–9 points. These scores indicate comparatively stronger selection, comparability, and outcome ascertainment, but do not eliminate residual confounding, exposure misclassification, or variation in outcome definitions. Findings from uncontrolled quality-improvement, cross-sectional, and service-evaluation designs were therefore interpreted with lower confidence. Detailed scores are shown in Table 2.

### 3.2. Mortality and Clinical Outcomes in Outborn Neonates

Among the 15 studies addressing outborn versus inborn outcomes, the majority (12 of 15) reported increased mortality among outborn neonates, particularly in very preterm and extremely preterm populations. Large population-based and cohort studies consistently indicated that birth outside tertiary perinatal centres is associated with higher mortality, with some studies reporting markedly elevated risks in the absence of timely referral or specialised care. In a propensity score-matched UK national cohort of extremely preterm infants, the adjusted odds ratio for in-hospital mortality among outborn versus inborn infants was 2.01 (95% CI 1.31–3.07), while a Japanese analysis of 15,842 VLBW singletons identified a significantly elevated risk of severe intraventricular haemorrhage among outborn infants (OR 3.45; 95% CI 1.16–10.3) [6,7,8,10,13,33].

In addition to mortality, 9 of the 15 studies reported increased rates of major neonatal complications among outborn infants, including severe intraventricular haemorrhage, necrotising enterocolitis, bronchopulmonary dysplasia, and late-onset sepsis [7,9,10,13]. However, these associations were not entirely consistent across all healthcare systems [8,32].

In well-organised regionalised networks, outcome differences between outborn and inborn infants were often attenuated or non-significant, suggesting that the observed risk is strongly influenced by system-level factors such as antenatal transfer practices, early stabilisation, and access to specialised neonatal care [8,32].

**Figure 2:**
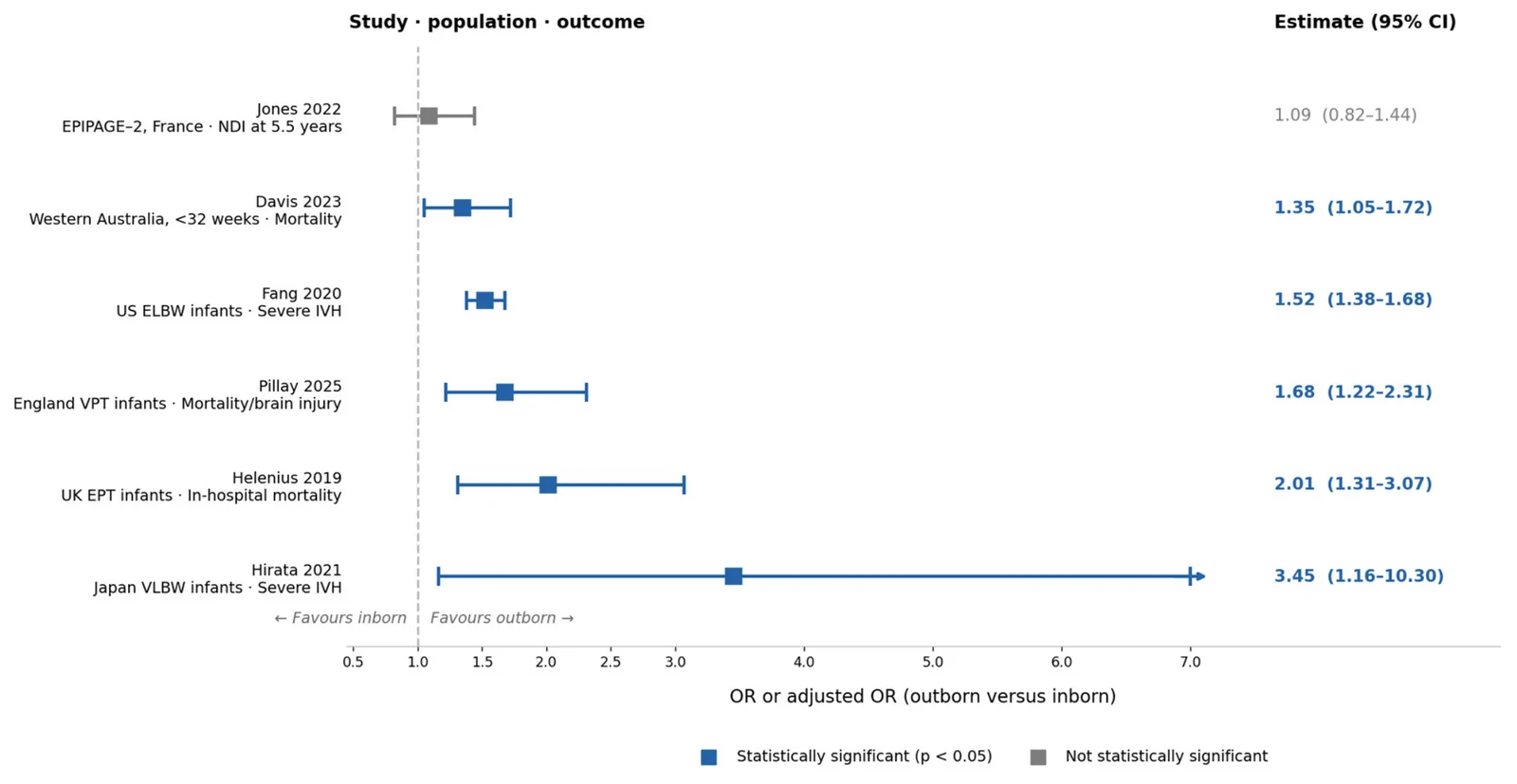
Effect estimates (OR or aOR, 95% CI) for mortality and severe morbidity in outborn versus inborn neonates, reproduced from Davis et al. [6], Helenius et al. [7], Pillay et al. [8], Fang et al. [10], Jones et al. [31], and Hirata et al. [33]. Populations and adjustments differed; no pooling was undertaken. Estimates are ordered by effect size. Hirata et al.’s upper confidence limit (10.30) was truncated at 7.0.

Most primary cohort studies reported higher mortality or major morbidity among outborn neonates, particularly in very or extremely preterm populations. However, effect magnitude varied by case-mix, confounder adjustment, regionalisation, and access to antenatal transfer. Studies from highly organised neonatal networks generally reported smaller or non-significant differences. Selected adjusted effect estimates are illustrated in Figure 2 without formal pooling.

### 3.3. Transport-Related Physiological Instability and Adverse Events

Across the primary studies that evaluated transport-related complications, clinically important physiological instability was reported repeatedly. Frequently described complications included hypothermia, respiratory deterioration, desaturation, hypoglycaemia, and haemodynamic instability [3,11,12,13,14,15,21,22,23,36].

Broader adverse-event profiles were described in both cohort studies and contextual reviews of neonatal transport, highlighting risks related to inadequate monitoring, delayed transfer, and non-specialised transport teams [4,20,36,39]. System-focused publications also emphasised variability in safety metrics and reporting practices [40,41].

Transport-related instability was reported particularly in resource-constrained settings [3,11,12,13,22,23], whereas studies of organised neonatal transport systems highlighted the potentially mitigating effects of standardised protocols and specialised teams [26,27,36,37,41].

Across primary studies, physiological instability during neonatal transport was reported most often in resource-limited or non-specialised systems. Hypothermia, respiratory deterioration, desaturation, hypoglycaemia, and haemodynamic instability were recurrent outcomes, although their definitions and ascertainment differed. Transport duration, modality, pre-departure stabilisation, team composition, and monitoring capacity appeared to modify risk. Air transport reduced travel time in remote settings but introduced additional vibration, noise, and pressure-related stressors. These sources of heterogeneity are detailed in Supplementary Table S2. The main findings across the evidence domains are summarised in Table 3.

### 3.4. Hypothermia and Thermal Management During Transport

Hypothermia was among the most consistently reported transport-associated complications. High admission prevalence was described particularly where standardised thermal protection was unavailable or inconsistently applied [13,14,15,24,25,42].

Hypothermia was associated with worse short-term outcomes in observational evidence, although the strength of association varied by population and study design [13,14,15,24,42].

Quality-improvement studies indicated that transport-related hypothermia is modifiable. Pre-warmed incubators, plastic wraps, thermal mattresses, continuous temperature monitoring, and structured thermal bundles were associated with lower admission hypothermia [13,15,24,25,42].

**Figure 3:**
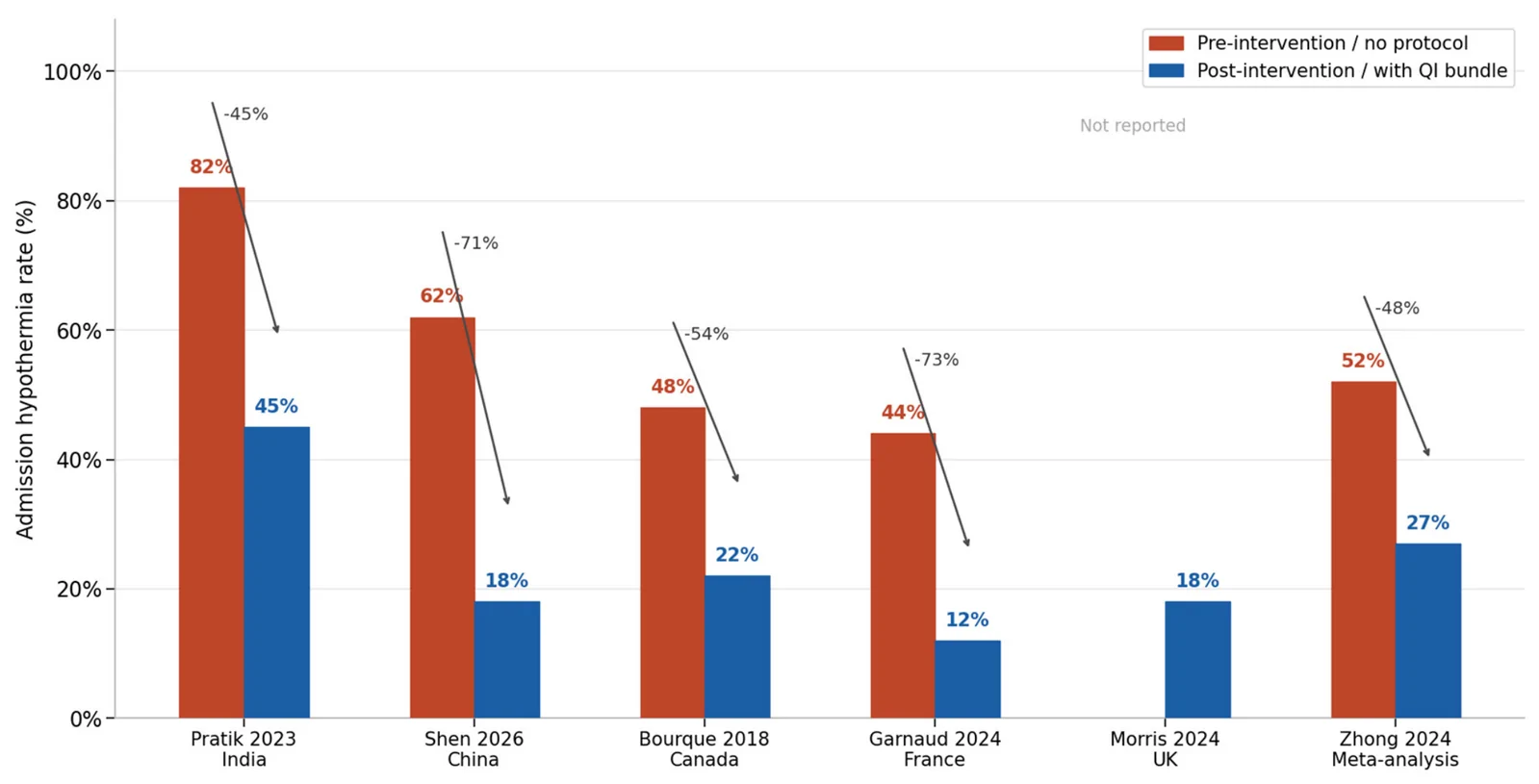
Admission hypothermia rates before and after study-specific thermal-management or quality-improvement interventions: Pratik et al. [13] (82% versus 45%; corresponding normothermia rates at NICU admission were 18% versus 55%, calculated as 100% minus the reported hypothermia rates), Shen et al. [43] (62% versus 18%), Bourque et al. [25] (48% versus 22%; the proportion of neonates admitted within the target range of 33–34 °C increased from 22% to 58%), Garnaud et al. [24] (44% versus 12%; the proportion reaching the target temperature on arrival increased from 32% to 100%), Morris et al. [15] (pre-intervention value not reported; 18% after intervention), and Zhong et al. [42] (52% versus 27%). Arrows indicate the relative reduction in hypothermia rates where both pre- and post-intervention values were available.

Evidence concerning admission hypothermia was more directionally consistent than evidence in other domains, although temperature thresholds, gestational-age criteria, and intervention bundles varied. A contextual systematic review and meta-analysis reported lower admission hypothermia after bundled interventions (pooled OR 0.31) [42]; this secondary estimate was used for context and was not combined with the primary studies in the present synthesis. Study-specific thermal-management outcomes from selected primary quality-improvement studies are illustrated in Figure 3.

### 3.5. Severity Scores and Prognostic Tools

Primary studies evaluated TRIPS, TRIPS-II, and other neonatal transport-scoring approaches. Collectively, this evidence supports physiology-based scores as practical markers of transport-related clinical risk [16,17,18,19,35,38]. The principal characteristics of TRIPS and TRIPS-II are summarised in Table 4.

Higher severity scores, and particularly worsening physiological status during transport, were associated with mortality and adverse neurodevelopmental outcomes [18,19]. TRIPS and TRIPS-II were also described as practical tools for risk stratification and transport-service benchmarking [16,17,18,19,35,38,41].

As highlighted in Table 4, the two instruments differ not only in scoring but in their intended clinical application. TRIPS was designed primarily as a physiological stability assessment tool for use during and immediately after transport, enabling serial monitoring throughout the transfer journey. TRIPS-II, by contrast, was optimised for rapid post-arrival risk stratification—a distinction with direct implications for how each score is best integrated into clinical workflows and transport programme audits. Importantly, TRIPS-II has been validated across broader patient populations, including both preterm and term outborn neonates in multiple international cohorts, extending its applicability beyond the extremely preterm infants in whom TRIPS was originally validated. In practice, the choice between instruments may be guided by clinical context: TRIPS supports detailed physiological monitoring during transfer, while TRIPS-II enables rapid post-arrival triage and quality benchmarking across transport programs. Both tools, however, share the fundamental value of translating complex physiological data into a single actionable index that supports risk communication between referring and receiving teams [18,19].

### 3.6. System-Level Factors, Organisation, and Quality of Neonatal Transport

System-level studies and transport-focused reviews indicated that outcomes after neonatal transfer are shaped by regionalisation, tertiary-care capacity, referral pathways, mobilisation time, interfacility communication, transport-team expertise, and consistent data collection [3,4,5,6,20,26,27,36,37,40,41,44].

Across system-level studies, neonatal transport outcomes varied according to the organisation of regional perinatal care, availability of specialised transport teams, transfer pathways, communication systems, and consistency of reporting practices. Studies conducted in highly regionalised systems generally reported narrower differences between outborn and inborn cohorts than studies from fragmented or resource-limited settings. Analysis of the UK Neonatal Transport Group national dataset over a ten-year period (2012–2021) demonstrated substantial regional variation in transport volume and outcome metrics, whilst also documenting measurable improvement in key indicators over time—underscoring the value of national-level data collection for benchmarking and quality improvement [44]. The principal modifiable transport- and system-related factors identified across the included studies are summarised in Table 5.

### 3.7. Overall Synthesis and Sources of Heterogeneity

Heterogeneity arose at several levels. Populations ranged from term neonates to extremely preterm or extremely low-birth-weight infants, and outborn status was defined variously by place of birth, postnatal transfer, or admission from another facility. Transport systems differed in regional organisation, team composition, pre-transport stabilisation, ground or air modality, distance, and available equipment. Definitions of BPD, NEC, sepsis, hypothermia, adverse events, and neurodevelopmental impairment were also inconsistent. Consequently, differences in reported outcomes may reflect both true clinical variation and measurement or classification differences.

Despite this heterogeneity, the direction of evidence was reasonably consistent for three findings: outborn very preterm infants often had poorer outcomes than inborn counterparts; hypothermia and physiological deterioration remained recurrent during transfer; and organised regional pathways, specialised teams, and structured stabilisation were associated with better transport quality. Confidence was lower for the magnitude of these associations and for their generalisability across healthcare systems.

## 4. Discussion

### 4.1. Summary of Main Findings

This review suggests that postnatal transfer from lower-capability facilities to tertiary or quaternary neonatal centres is generally associated with higher mortality and morbidity than inborn care, particularly among very and extremely preterm infants. However, this association was attenuated in some well-regionalised systems and should not be interpreted as a uniform causal effect of transport.

The transport interval remains clinically vulnerable: hypothermia, respiratory or haemodynamic instability, and adverse events were repeatedly reported, especially where pre-transport stabilisation, specialist staffing, or equipment was limited. TRIPS and TRIPS-II provide practical physiological risk measures, but differences in case-mix, outcome definitions, and study quality limit uniform quantitative conclusions across settings.

### 4.2. Outborn Versus Inborn Status in the Era of Regionalised Perinatal Care

The regionalisation of perinatal care aims to concentrate high-risk births in tertiary centres, reducing the need for postnatal transfer [1,2]. Nevertheless, a substantial proportion of very preterm and high-risk infants are still born in non-tertiary facilities, especially in regions with geographic barriers, shortages of NICU beds, or incomplete referral networks [3,4,5]. Contemporary cohorts from Western Australia, England, and Japan consistently report higher mortality and severe morbidity in outborn infants compared with inborn infants, even after adjusting for gestational age and illness severity [6,7,8,10,13]. These findings reinforce the principle that place of birth remains a significant determinant of outcome for highly vulnerable neonates.

However, the association between outborn status and poor outcome is not uniform across all health systems. In highly regionalised networks with strong antenatal risk identification, timely in utero transfer, and standardised pre- and postnatal protocols, some studies report attenuated or absent differences between inborn and outborn infants [8,32]. The most compelling evidence comes from the EPIPAGE-2 prospective cohort, in which, after matching for gestational age and antenatal corticosteroid coverage, no statistically significant difference in survival without moderate-to-severe neurodevelopmental impairment was observed between outborn and inborn preterm infants at 5.5 years of age (OR 1.09; 95% CI 0.82–1.44; *p* = 0.54) [32]. This suggests that outborn status is not an immutable risk factor, but rather a proxy for upstream determinants such as delayed referral, limited antenatal steroid use, inadequate delivery-room care, and suboptimal stabilisation before transfer. These findings are, however, specific to a highly regionalised French perinatal system and should not be extrapolated uncritically to settings where antenatal steroid coverage and in utero transfer rates are substantially lower. For clinical practice and policy, this underscores the dual priority of maximising in utero transfer whenever feasible and ensuring that inevitable postnatal transfers are performed under optimal conditions [3,4,5,8,32].

### 4.3. Transport-Phase Risks: Hypothermia and Physiologic Instability

Admission hypothermia is one of the most consistent and modifiable complications of neonatal transfer [13,14,15,24,25,42]. Quality-improvement studies show that pre-warmed incubators, plastic wraps, hats, continuous temperature monitoring, and staff training can reduce hypothermia at arrival [13,15,24,25,42]. Severe hypothermia may cause arrhythmia, respiratory failure, and circulatory collapse and requires management beyond the capability of most transport environments [45]. A regional collaborative published after the prespecified search window provides additional supportive quality-improvement context [43], but it was not counted among the 44 retained publications or used in the primary synthesis.

Beyond temperature, transported infants are vulnerable to respiratory deterioration, hypotension, hypoglycaemia, and other markers of physiologic instability [3,15,16,31]. These events are more frequent when transport teams lack neonatal-specific training or when transport is performed by general emergency services rather than dedicated neonatal transport teams [35,36]. Quantitative evidence of this effect was provided by a retrospective comparison from Western Australia, in which neonatal specialist teams generated significantly fewer unintended clinical events compared with non-specialist teams (57% vs. 77% of transports), and demonstrated complete elimination of endotracheal tube-related adverse events (0% vs. 30%) [35]. The literature indicates that high-quality pre-transport stabilisation and continuous monitoring during transfer are central to mitigating these risks.

Severity-of-illness scores such as TRIPS and TRIPS-II summarise temperature, respiratory status, blood pressure, and responsiveness into a single index [18,19]. Higher TRIPS scores at arrival, and particularly worsening TRIPS during transport, are strongly associated with early mortality and adverse neurodevelopmental outcomes in extremely preterm infants, with each unit increase in TRIPS score associated with an adjusted OR of 1.3–1.5 for death or neurodevelopmental impairment at 18–24 months [20]. From a systems perspective, TRIPS and TRIPS-II can serve as both clinical tools and quality indicators for transport services, enabling benchmarking and targeted improvement [18,19,20].

### 4.4. Duration, Distance, and Mode of Transport

Transport duration and distance are plausible risk modifiers, but the evidence is context-dependent. A Swiss cohort associated longer transport with greater physiological instability at arrival, particularly in VLBW and ELBW infants [21]. Evidence from resource-constrained settings suggests that prolonged journeys may amplify risk when stabilisation and monitoring are suboptimal [12,22,23].

No included study established a strict dose–response relationship between each additional minute or kilometre and clinical risk. Transport time interacts with illness severity, team expertise, equipment, and environmental conditions [20,21,22,36]. Air transport may shorten remote retrievals but adds vibration, noise, and pressure-related stressors [39]. Process redesign has nevertheless shown that shorter mobilisation times for urgent neonatal transfers are achievable [27].

### 4.5. Role of Specialised Neonatal Transport Teams and System Organisation

A recurrent finding was that specialised neonatal transport teams were associated with fewer adverse events and better physiological stability than non-specialised services. Nevertheless, most supporting studies were observational or service evaluations, and residual confounding by case selection, regional resources, and referral practices remains possible.

System-level analyses and contextual reviews indicate that transport outcomes are shaped by the availability of beds in tertiary and quaternary neonatal centres, regional referral protocols, communication between sending and receiving hospitals, and standardised stabilisation and transport guidance [3,6,20,40,41,44]. The international NeoTemplate consensus framework identified 37 core variables for standardised reporting of neonatal intensive care transport, supporting meaningful cross-system comparison [40]. Where perinatal regionalisation is well implemented, clear triage and transfer pathways may mitigate the disadvantage associated with outborn status. Conversely, fragmented systems with ad hoc decision-making, scarce specialised teams, and limited neonatal-specific equipment were associated with poorer outcomes [3,6,12,22,23].

For policy makers and clinicians, the evidence supports investment in coordinated neonatal transport systems, including trained teams, neonatal-specific equipment, standardised stabilisation protocols, communication pathways, and continuous audit. In low- and middle-income settings, feasible priorities may include thermal bundles, structured pre-referral communication, minimum monitoring standards, competency-based training, and regional referral criteria; implementation should be adapted to local resources rather than directly transferring high-income service models.

### 4.6. Long-Term Outcomes and Neurodevelopmental Vulnerability

Although limited, follow-up data suggest that transport-related vulnerability may extend beyond the neonatal period. Cohorts from Western Australia, Japan, and France evaluated neurodevelopmental outcomes among outborn or transferred infants [6,31,34]. Among extremely preterm infants, worsening TRIPS during transport was associated with death or neurodevelopmental impairment at 18–24 months [18]. In the matched EPIPAGE-2 analysis, adjustment for gestational age and antenatal corticosteroid exposure attenuated the apparent outborn disadvantage, supporting cautious interpretation of transport as one component of a broader care pathway [31].

These findings are biologically plausible: severe hypothermia, hypotension, and respiratory instability during a critical period of brain vulnerability may contribute to white matter injury and long-term functional deficits [7,10,20]. However, the available studies cannot fully disentangle the effects of transport-related events from confounding factors such as antenatal care, perinatal asphyxia, illness severity at birth, and socioeconomic determinants [6,10,14,32]. Therefore, outborn status and transport-related instability should be viewed as risk markers rather than isolated causal factors, underscoring the need for comprehensive approaches spanning antenatal, perinatal, and postnatal care.

### 4.7. Strengths, Limitations, and Research Gaps

This review integrates contemporary evidence across mortality, morbidity, transport-phase instability, severity scoring, and system organisation. PRISMA 2020 reporting, SWiM-informed narrative synthesis, explicit separation of primary from contextual evidence, and structured risk-of-bias assessment support transparency (Table S3).

Several limitations require consideration. The protocol was not prospectively registered. Only English-language publications were eligible, and grey literature was not systematically searched, creating potential language and dissemination bias. Most primary studies were observational and vulnerable to residual confounding, selection bias, and inconsistent adjustment for gestational age, birth weight, antenatal care, and illness severity. Definitions of outborn status, transport exposures, BPD, NEC, sepsis, hypothermia, adverse events, and neurodevelopmental impairment varied across studies. This heterogeneity prevented meta-analysis and limited formal assessment of small-study effects. Positive quality-improvement findings and studies showing large outborn–inborn differences may be more likely to be published, potentially inflating apparent effects. Evidence from low- and middle-income settings and long-term follow-up remained limited. The record-level screening log was no longer available at revision; therefore, the full-text-stage counts were reconstructed from the contemporaneous manuscript summary, and no inter-reviewer agreement statistic was retrospectively calculated.

Overall confidence was highest for the recurrent direction of association between outborn status and adverse outcomes in very preterm infants and for reductions in admission hypothermia after structured thermal interventions. Confidence was lower for the magnitude and causal interpretation of these effects, for comparisons of specialist versus non-specialist teams, and for long-term neurodevelopmental outcomes because the evidence was heterogeneous, predominantly observational, and unevenly adjusted for key confounders.

Future research should prioritise:

- Prospective multicentre registries of neonatal transport incorporating standardised physiological variables and TRIPS/TRIPS-II scores, enabling meaningful cross-centre and cross-national benchmarking;
- High-quality comparative studies of specialised versus non-specialised transport teams with sufficient statistical power to detect clinically meaningful differences in adverse events, mortality, and morbidity;
- Rigorous evaluation of specific interventions—thermal bundles, standardised stabilisation protocols, telemedicine support to referring centres—using pre-specified outcome measures and controlled designs;
- Long-term prospective follow-up of matched outborn and inborn extremely preterm infants to clarify the relative contributions of antenatal, perinatal, and transport-related factors to neurodevelopmental outcomes;
- International harmonisation of transport data collection, building on the NeoTemplate consensus framework, to enable cross-national epidemiological analyses and identification of best practices across diverse healthcare systems [40].

### 4.8. Implications for Romanian Clinical Practice

The findings are relevant to healthcare systems in which perinatal regionalisation is still consolidating, including Romania. The Romanian cohort by Ognean et al. evaluated mortality prediction among 418 outborn neonates [35], underscoring the need for timely referral and reproducible severity assessment. TRIPS and TRIPS-II have not yet been prospectively validated in Romanian transport cohorts; implementation studies could evaluate their usefulness while aligning registry variables with the NeoTemplate framework [16,17,18,19,35,40]. Thermal-management bundles, structured transport-team training, and a national neonatal transport registry are pragmatic priorities, but should be evaluated prospectively in the Romanian care context.

## 5. Conclusions

Predominantly observational evidence suggests that postnatal neonatal transfer to tertiary or quaternary centres is associated with higher mortality and morbidity, particularly among very preterm infants. The association varies across regional systems and appears to reflect both baseline vulnerability and differences in antenatal referral, stabilisation, transport conditions, and access to specialist care.

Hypothermia, cardiorespiratory instability, and adverse events remain important and potentially modifiable transport-phase risks. Specialised teams, thermal-management bundles, structured communication, and standardised transport pathways were associated with better physiological stability, although stronger comparative evidence is required before causal effects can be established.

Future research should prioritise prospective multicentre registries, harmonised outcome definitions, comparative evaluation of transport-team models, validation of TRIPS/TRIPS-II in resource-variable settings, and long-term neurodevelopmental follow-up. Standardised data collection may improve benchmarking while preserving the need for context-specific interpretation.

## Figures and Tables

**Table 1:** Main characteristics of retained publications by thematic domain.

Thematic Domain	No. of Retained Publications	Main Study Types	Principal Outcomes or Focus
Outborn vs. inborn outcomes and regionalisation	15	Cohort studies, population-based studies	Mortality, severe brain injury, NEC, BPD, sepsis, neurodevelopment, and the effect of birth setting
Transport systems, safety, reporting, and organisation	11	Primary cohort and quality-improvement studies; contextual systematic reviews	Safety metrics, reporting heterogeneity, transport systems, referral pathways, and team mobilisation
Transport complications, referral, and stabilisation	5	Observational cohort studies	Pre-transport condition, referral challenges, adverse events, intrahospital/interfacility instability
Hypothermia and thermal management	9	Observational studies, quality-improvement studies	Admission hypothermia, thermal interventions, cooling/temperature management during transport
Severity scores and prognostic tools	4	Score development, validation, and cohort studies	TRIPS, TRIPS-II, transport-related risk prediction, mortality, neurodevelopment

**Table 2:** Newcastle–Ottawa Scale quality assessment of primary observational studies included in the review.

Ref.	Study	Design	Selection (0–4)	Comparability (0–2)	Outcome (0–3)	Total NOS (0–9)	Quality Category
[6]	Davis et al., 2023	Retrospective population-based cohort	4	2	2	8	High
[7]	Helenius et al., 2019	Observational cohort with propensity score matching	4	2	3	9	High
[8]	Pillay et al., 2025	National cohort study	4	2	2	8	High
[9]	Chen et al., 2021	Retrospective cohort study	3	2	2	7	High
[10]	Fang et al., 2020	Retrospective cohort study	3	2	3	8	High
[11]	Singh et al., 2021	Prospective observational cohort	3	1	3	7	High
[12]	Yeshaneh et al., 2021	Observational cohort study	3	1	3	7	High
[18]	Grass et al., 2020	Cohort study	4	2	2	8	High
[19]	Peng et al., 2024	Real-world observational cohort	3	2	2	7	High
[21]	Schwarz et al., 2025	Retrospective single-centre cohort	3	1	3	7	High
[22]	Tette et al., 2020	Cross-sectional observational study	N/A	N/A	N/A	N/A	Not assessed with standard NOS
[23]	Meshram and Thangavelu, 2025	Prospective observational cohort	3	1	3	7	High
[31]	Jones et al., 2022	Prospective matched cohort	4	2	3	9	High
[32]	Balog et al., 2025	Observational cohort study	3	2	2	7	High
[33]	Hirata et al., 2021	National cohort study	4	2	2	8	High
[34]	Hirata et al., 2022	Prospective birth cohort	4	2	2	8	High
[35]	Ognean et al., 2023	Retrospective cohort study	3	2	2	7	High
[36]	Gardiner et al., 2024	Retrospective comparative cohort	3	1	3	7	High
[37]	Marsinyach Ros et al., 2020	Retrospective cohort/service evaluation	3	1	3	7	High
[38]	Qu et al., 2022	Retrospective cohort study	3	2	2	7	High

Abbreviations: NOS, Newcastle–Ottawa Scale. The NOS was applied to observational cohort and case–control studies. Systematic reviews, methodological papers, Delphi consensus studies, quality-improvement reports, and cross-sectional studies were not assessed using the standard NOS framework. Quality categories were defined as follows: low quality, 0–4 points; moderate quality, 5–6 points; and high quality, 7–9 points.

**Table 3:** Summary of the main findings across the included evidence base.

Evidence Domain	Consistency of Evidence	Main Findings	Representative References
Mortality in outborn neonates	Consistent across most cohort studies	Outborn status was associated with increased mortality, particularly among very preterm infants	[6,7,8,9,10,11,12,13]
Major neonatal morbidities	Frequently reported across observational studies	Higher rates of IVH, NEC, BPD, and sepsis were reported in multiple cohorts	[7,8,9,10,11,12,13]
Transport-related physiologic instability	Consistently reported	Hypothermia, respiratory deterioration, and haemodynamic instability were common during transport	[14,15,16,17,22]
Hypothermia and thermal management	Strongly consistent	Hypothermia was identified as a frequent and potentially modifiable complication	[15,16,17]
Severity assessment tools	Consistently supported	TRIPS and TRIPS-II demonstrated prognostic utility during neonatal transport	[18,19,20,21]
Organizational and system-level determinants	Consistent across transport-system studies	Specialized transport teams and regionalised care systems were associated with improved transport quality	[4,22,23,24,25,26,27]

**Table 4:** Comparative characteristics, validation evidence, and clinical applications of TRIPS and TRIPS-II neonatal transport severity scores—Panel A: Technical and scoring characteristics; Panel B: Clinical applications and quality improvement utility.

Characteristic	TRIPS	TRIPS—II
PANEL A—Technical and Scoring Characteristics		
Original publication	Lee et al., 2001 [16]	Lee et al., 2013 [17]
Primary objective	Assessment of physiologic stability during neonatal transport [16]	Simplified prediction of mortality risk during neonatal transport [17]
Core physiologic domains	Temperature, respiratory status, blood pressure, and neurological responsiveness [16]	Temperature, respiratory status, blood pressure, and neurological responsiveness [17]
Scoring structure	4 domains; detailed multi-threshold scoring; longer to apply [16]	Same 4 domains; minor scoring adjustment for O_2_ supplementation; rapid bedside application [17]
Score interpretation	Higher score = greater physiological instability	Higher score = higher mortality risk
Population validated	Preterm infants; Canadian Neonatal Network cohort [18]	Preterm and term outborn neonates; multiple international cohorts [17,21]
Principal outcomes in validation studies	Mortality, physiologic deterioration, and neurodevelopmental outcomes [18,20]	Mortality prediction, transport benchmarking, and severity adjustment [17,21]
PANEL B—Clinical Applications and Quality Improvement Utility		
Intended clinical use	Serial physiological monitoring during and immediately after transport; enables assessment of transport-related deterioration [16,18]	Rapid post-arrival risk stratification; bedside triage and mortality prediction [17,19]
Quality indicator use	Transport programme benchmarking; serial assessment during transfer [25,26,27]	Bedside triage; mortality prediction; quality improvement audit [17,21,25,26]
Utility for QI initiatives	Frequently used as a transport quality indicator and benchmarking tool [25,26,27]	Frequently incorporated into quality-improvement and service-evaluation frameworks [25,26,27]
Strengths	Broad physiologic characterisation; extensive validation history; association with mortality and neurodevelopmental outcomes [18,20,25]	Simpler structure; easier implementation; strong prognostic performance in routine clinical settings [17,21,26]
Limitations	Greater complexity and training requirements; more detailed data collection needed; validated primarily in high-income settings [18,25]	Reduced physiologic granularity compared with TRIPS; limited evidence in resource-constrained environments [17,21]
Relevance to contemporary systems	Supports monitoring of transport-related physiologic instability and benchmarking of transport performance [18,20,25,26]	Supports risk adjustment, service evaluation, and outcome prediction within regionalised transport networks [17,21,25,26]

Abbreviations: QI, quality improvement. Sources: TRIPS [16,18]; TRIPS-II [17,19]. The “Metabolic stability” criterion present in some prior descriptions of TRIPS-II has been omitted, as it does not form part of the published scoring algorithm. Panel A presents technical and scoring characteristics; Panel B presents clinical applications and quality improvement utility.

**Table 5:** Main modifiable transport- and system-related factors identified in the included studies.

Factor	Clinical Relevance	Likely Effect on Outcomes
Timely in utero transfer when feasible	Reduces the need for postnatal transport	Associated with lower mortality and major morbidity by avoiding unstable transfer after birth [1,2,8]
Pre-transport stabilisation	Central determinant of transport safety	Reduces physiological deterioration during transfer
Specialized neonatal transport teams	Improves monitoring and response capability	Associated with fewer adverse events and better stability
Continuous thermal management	Prevents hypothermia	Associated with improved thermal stability at arrival [13,15,24,25,42]
Standardized transport protocols	Reduces practice variability	Improves consistency, safety, and auditability
Rapid team mobilization and coordinated referral	Reduces delays to tertiary care	Associated with decreased deterioration before and during transfer [26,27,35,36]
Consistent data collection and reporting	Enables benchmarking and quality improvement	Improves comparability and supports system-level optimization

## Data Availability

No new data were created or analyzed in this study. Data sharing is not applicable to this article.

## Supplementary Materials

The following supporting information can be downloaded at: https://www.mdpi.com/article/10.3390/children13081064/s1, Table S1: Database-specific search strategies; Table S2: Structured assessment of clinical and methodological heterogeneity; Table S3: PRISMA 2020 Checklist.
