# Supplementary material for: Neonatal Interfacility Transport to Tertiary and Quaternary Centres: Clinical Outcomes and System-Level Determinants—A Systematic Review
Source: Children (Basel). 2026 Aug 10;13(8):1064. doi: 10.3390/children13081064 (PMC13511184; doi:10.3390/children13081064)
Supplement: Supplementary file 1 [file children-13-01064-s001.zip › children-4432305-supplementary-Table S3. PRISMA 2020 Checklist.pdf]

PRISMA 2020 Checklist

Section and Topic	Item #	Checklist item	Location where item is reported
**TITLE**			
Title	1	Identify the report as a systematic review.	Title page (the title identifies the report as a systematic review).
**ABSTRACT**			
Abstract	2	See the PRISMA 2020 for Abstracts checklist.	Abstract.
**INTRODUCTION**			
Rationale	3	Describe the rationale for the review in the context of existing knowledge.	Section 1, Introduction, paragraphs 1–3.
Objectives	4	Provide an explicit statement of the objective(s) or question(s) the review addresses.	Section 1, Introduction, final paragraph.
**METHODS**			
Eligibility criteria	5	Specify the inclusion and exclusion criteria for the review and how studies were grouped for the syntheses.	Sections 2.3, Eligibility Criteria, and 2.7, Outcomes and Synthesis Domains.
Information sources	6	Specify all databases, registers, websites, organisations, reference lists and other sources searched or consulted to identify studies. Specify the date when each source was last searched or consulted.	Section 2.4, Information Sources and Search Strategy. The last search date is not stated separately for each source.
Search strategy	7	Present the full search strategies for all databases, registers and websites, including any filters and limits used.	Section 2.4, Information Sources and Search Strategy (full strategies and limits for PubMed/MEDLINE, Embase, Scopus, Web of Science, Cochrane Library, and Google Scholar).
Selection process	8	Specify the methods used to decide whether a study met the inclusion criteria of the review, including how many reviewers screened each record and each report retrieved, whether they worked independently, and if applicable, details of automation tools used in the process.	Section 2.5, Study Selection. Partially reported: the number of reviewers, independent screening, and disagreement-resolution procedures are not stated.
Data collection process	9	Specify the methods used to collect data from reports, including how many reviewers collected data from each report, whether they worked independently, any processes for obtaining or confirming data from study investigators, and if applicable, details of automation tools used in the process.	Section 2.6, Data Extraction. Partially reported: the number of reviewers, independent extraction/verification, and contact with study investigators are not stated.
Data items	10a	List and define all outcomes for which data were sought. Specify whether all results that were compatible with each outcome domain in each study were sought (e.g. for all measures, time points, analyses), and if not, the methods used to decide which results to collect.	Sections 2.2, Review Question and PEO Framework, and 2.7, Outcomes and Synthesis Domains.
	10b	List and define all other variables for which data were sought (e.g. participant and intervention characteristics, funding sources). Describe any assumptions made about any missing or unclear information.	Section 2.6, Data Extraction. Assumptions for missing or unclear information are not stated.
Study risk of bias assessment	11	Specify the methods used to assess risk of bias in the included studies, including details of the tool(s) used, how many reviewers assessed each study and whether they worked independently, and if applicable, details of automation tools used in the process.	Section 2.8, Risk-of-Bias and Methodological Appraisal. Partially reported: the number of assessors and whether assessments were independent are not stated.
Effect measures	12	Specify for each outcome the effect measure(s) (e.g. risk ratio, mean difference) used in the synthesis or presentation of results.	Not applicable. No meta-analysis or pooled effect estimates were performed; findings were synthesized narratively (Sections 2.1 and 2.7).
Synthesis methods	13a	Describe the processes used to decide which studies were eligible for each synthesis (e.g. tabulating the study intervention characteristics and comparing against the planned groups for each synthesis (item #5)).	Sections 2.3, Eligibility Criteria, 2.6, Data Extraction, and 2.7, Outcomes and Synthesis Domains.
	13b	Describe any methods required to prepare the data for presentation or synthesis, such as handling of missing summary statistics, or data conversions.	Not applicable/not reported. No data conversions or preparation of summary statistics were described.
	13c	Describe any methods used to tabulate or visually display results of individual studies and syntheses.	Section 2.7; Figure 1; Tables 1 and 2.

Section and Topic	Item #	Checklist item	Location where item is reported
	13d	Describe any methods used to synthesize results and provide a rationale for the choice(s). If meta-analysis was performed, describe the model(s), method(s) to identify the presence and extent of statistical heterogeneity, and software package(s) used.	Sections 2.1, 2.7, and 2.9. Narrative synthesis was used because of clinical and methodological heterogeneity; no meta-analysis was performed.
	13e	Describe any methods used to explore possible causes of heterogeneity among study results (e.g. subgroup analysis, meta-regression).	Not applicable. No subgroup analysis, meta-regression, or other formal investigation of heterogeneity was conducted.
	13f	Describe any sensitivity analyses conducted to assess robustness of the synthesized results.	Not applicable. No sensitivity analyses were conducted.
Reporting bias assessment	14	Describe any methods used to assess risk of bias due to missing results in a synthesis (arising from reporting biases).	Not reported. No formal assessment of reporting bias or bias due to missing results is described.
Certainty assessment	15	Describe any methods used to assess certainty (or confidence) in the body of evidence for an outcome.	Section 2.9, Strength of Inference. Certainty was judged narratively; no formal certainty framework is reported.
**RESULTS**			
Study selection	16a	Describe the results of the search and selection process, from the number of records identified in the search to the number of studies included in the review, ideally using a flow diagram.	Section 3.1, Study Selection, and Figure 1 (PRISMA 2020 flow diagram).
	16b	Cite studies that might appear to meet the inclusion criteria, but which were excluded, and explain why they were excluded.	Section 3.1 reports categories and reasons for full-text exclusion. Individual apparently eligible excluded studies are not cited.
Study characteristics	17	Cite each included study and present its characteristics.	Section 3.2, General Characteristics of the Included Studies, and Table 1.
Risk of bias in studies	18	Present assessments of risk of bias for each included study.	Not reported for each included study. The appraisal method is described in Section 2.8.
Results of individual studies	19	For all outcomes, present, for each study: (a) summary statistics for each group (where appropriate) and (b) an effect estimate and its precision (e.g. confidence/credible interval), ideally using structured tables or plots.	Table 1 and Sections 3.4–3.11. Findings are presented narratively; study-level effect estimates and precision measures are generally not applicable/not reported.
Results of syntheses	20a	For each synthesis, briefly summarise the characteristics and risk of bias among contributing studies.	Sections 3.2 and 3.3; Tables 1 and 2. Study characteristics are summarized, but risk-of-bias findings by synthesis are not presented.
	20b	Present results of all statistical syntheses conducted. If meta-analysis was done, present for each the summary estimate and its precision (e.g. confidence/credible interval) and measures of statistical heterogeneity. If comparing groups, describe the direction of the effect.	Not applicable. No statistical synthesis or meta-analysis was conducted.
	20c	Present results of all investigations of possible causes of heterogeneity among study results.	Not applicable. No formal investigation of causes of heterogeneity was conducted.
	20d	Present results of all sensitivity analyses conducted to assess the robustness of the synthesized results.	Not applicable. No sensitivity analyses were conducted.
Reporting biases	21	Present assessments of risk of bias due to missing results (arising from reporting biases) for each synthesis assessed.	Not reported. No assessment of reporting bias due to missing results is presented.
Certainty of evidence	22	Present assessments of certainty (or confidence) in the body of evidence for each outcome assessed.	Section 2.9 and Table 2. Strength of evidence is presented narratively rather than with a formal certainty framework.
**DISCUSSION**			
Discussion	23a	Provide a general interpretation of the results in the context of other evidence.	Sections 4.1, 4.2, and 4.7.
	23b	Discuss any limitations of the evidence included in the review.	Section 4.6, Strengths and Limitations.
	23c	Discuss any limitations of the review processes used.	Section 4.6, Strengths and Limitations.

PRISMA 2020 Checklist

Section and Topic	Item #	Checklist item	Location where item is reported
	23d	Discuss implications of the results for practice, policy, and future research.	Sections 4.4, 4.5, and 5, Conclusions.
**OTHER INFORMATION**			
Registration and protocol	24a	Provide registration information for the review, including register name and registration number, or state that the review was not registered.	Section 2.1 states that the review protocol was not prospectively registered.
	24b	Indicate where the review protocol can be accessed, or state that a protocol was not prepared.	Section 2.1 states that a protocol was developed before final data extraction; its accessibility is not reported.
	24c	Describe and explain any amendments to information provided at registration or in the protocol.	Not reported. No amendments to the protocol are described.
Support	25	Describe sources of financial or non-financial support for the review, and the role of the funders or sponsors in the review.	Funding statement.
Competing interests	26	Declare any competing interests of review authors.	Conflicts of Interest statement.
Availability of data, code and other materials	27	Report which of the following are publicly available and where they can be found: template data collection forms; data extracted from included studies; data used for all analyses; analytic code; any other materials used in the review.	Data Availability Statement. It states that no new data were created or analysed and that data sharing is not applicable.

From: Page MJ, McKenzie JE, Bossuyt PM, Boutron I, Hoffmann TC, Mulrow CD, et al. The PRISMA 2020 statement: an updated guideline for reporting systematic reviews. BMJ 2021;372:n71. doi: 10.1136/bmj.n71.
This work is licensed under CC BY 4.0. To view a copy of this license, visit https://creativecommons.org/licenses/by/4.0/
