# Supplementary material for: Neonatal Interfacility Transport to Tertiary and Quaternary Centres: Clinical Outcomes and System-Level Determinants—A Systematic Review
Source: Children (Basel). 2026 Aug 10;13(8):1064. doi: 10.3390/children13081064 (PMC13511184; doi:10.3390/children13081064)
Supplement: Supplementary file 1 [file children-13-01064-s001.zip › children-4432305-supplementary-Tables S1 and S2.pdf]

Supplementary Materials

Neonatal Interfacility Transport to Tertiary and Quaternary Centres: Clinical Outcomes and System-Level Determinants—A Systematic Review

Supplementary Table S1. Database-specific search strategies

The search was initially conducted in September 2025 and updated in December 2025. The documented strategies were rerun in July 2026 to verify reproducibility and consolidate the record set; the eligibility end date remained December 2025. Vocabulary and syntax were adapted to each platform. The table reports the complete reproducibility syntax for the four core concepts: neonatal population, interfacility transport/retrieval, tertiary or quaternary neonatal care, and clinical outcomes. The audit identified 2,456 records before deduplication and 1,371 unique records after removal of 1,085 duplicates. Database indexing and interface updates may lead to small differences in retrieved counts when the strategies are rerun.

Database/platform	Search strategy	Limits
MEDLINE via PubMed	("Infant, Newborn"[MeSH] OR neonate*[tiab] OR newborn*[tiab]) AND ("Transportation of Patients"[MeSH] OR transport*[tiab] OR transfer*[tiab] OR retrieval[tiab] OR "neonatal retrieval"[tiab] OR "transport service"[tiab] OR "transport team"[tiab] OR "interhospital transport"[tiab] OR "interfacility transport"[tiab] OR "aeromedical transport"[tiab]) AND ("Intensive Care Units, Neonatal"[MeSH] OR "level III"[tiab] OR "level IV"[tiab] OR tertiary[tiab] OR quaternary[tiab] OR "regionalized perinatal care"[tiab]) AND (mortality[tiab] OR morbidity[tiab] OR outcome*[tiab] OR "Treatment Outcome"[MeSH]) AND ("2010/01/01"[Date - Publication] : "2025/12/31"[Date - Publication])	English; 2010–2025; audit export n = 953
Scopus	TITLE-ABS-KEY((neonat* OR newborn*) AND (transport* OR transfer* OR retrieval OR "neonatal retrieval" OR "transport service" OR "transport team" OR "interhospital transport" OR "interfacility transport" OR "aeromedical transport") AND ("neonatal intensive care" OR "level III" OR "level IV" OR tertiary OR quaternary OR "regionalized perinatal care") AND (mortality OR morbidity OR outcome*)) AND PUBYEAR > 2009 AND PUBYEAR < 2026	English; 2010–2025; audit export n = 652
Web of Science Core Collection	TS=((neonat* OR newborn*) AND (transport* OR transfer* OR retrieval OR "neonatal retrieval" OR "transport service" OR "transport team" OR "interhospital transport" OR "interfacility transport" OR "aeromedical transport") AND ("neonatal intensive care" OR "level III" OR "level IV" OR tertiary OR quaternary OR "regionalized perinatal care") AND (mortality OR morbidity OR outcome*))	English; 2010–2025; audit export n = 758
Cochrane Library	([mh "Infant, Newborn"] OR neonate*:ti,ab,kw OR newborn*:ti,ab,kw) AND ([mh "Transportation of Patients"] OR transport*:ti,ab,kw OR transfer*:ti,ab,kw OR retrieval:ti,ab,kw OR "neonatal retrieval":ti,ab,kw OR "transport service":ti,ab,kw OR "transport team":ti,ab,kw OR "interhospital transport":ti,ab,kw OR "interfacility transport":ti,ab,kw OR "aeromedical transport":ti,ab,kw) AND ([mh "Intensive Care Units, Neonatal"] OR "level III":ti,ab,kw OR "level IV":ti,ab,kw OR tertiary:ti,ab,kw OR quaternary:ti,ab,kw OR "regionalized perinatal care":ti,ab,kw) AND (mortality:ti,ab,kw OR morbidity:ti,ab,kw OR outcome*:ti,ab,kw OR [mh "Treatment Outcome"])	English; 2010–2025; audit export n = 93

Supplementary Table S2. Structured assessment of clinical and methodological heterogeneity

The categories below were used to interpret variability across studies in accordance with SWiM principles. They were not treated as statistically pooled subgroups because the available studies differed simultaneously across several domains.

Domain	Observed or expected variation across included studies	Implication for comparability	Approach in the narrative synthesis
**Population and case mix**	All neonates versus VLBW/ELBW, very preterm, extremely preterm, term, or diagnosis-specific cohorts; different baseline illness severity.	Baseline risks differ substantially; crude event rates and unadjusted outborn–inborn comparisons are not directly exchangeable.	Grouped findings by population; prioritised adjusted estimates and interpreted magnitude cautiously.
**Outborn/transfer exposure**	Birth outside the receiving tertiary centre, any postnatal interhospital transfer, early transfer, or transfer within regional networks.	Exposure groups may represent different clinical pathways and referral delays.	Used study-specific definitions and avoided treating all outborn categories as identical.
**Neonatal-care level**	AAP Levels III/IV, tertiary/quaternary centres, and country-specific functional designations.	Identical labels do not guarantee identical staffing, surgery, subspecialty, or referral capabilities.	Used tertiary/quaternary or locally equivalent terminology and interpreted findings within each system.
**Regional organisation and setting**	Highly regionalised networks versus fragmented referral systems; high-income versus resource-limited settings; geographic and bed-capacity constraints.	Antenatal transfer, referral timeliness, equipment, and access to specialised teams can modify outcomes.	Synthesised system-level findings separately and avoided direct transfer of high-income models to all settings.
**Stabilisation and team model**	Variable thermal bundles, airway management, monitoring, pre-departure checklists, neonatal-specialist staffing, and communication protocols.	Observed differences may reflect the complete care pathway rather than transport alone.	Interpreted team effects as associations and highlighted residual confounding and implementation context.
**Transport duration, distance, and modality**	Short urban ground transfer, prolonged regional transfer, fixed-wing or rotary-wing aeromedical retrieval; inconsistent time origins.	Distance and duration interact with severity, stabilisation, weather, equipment, and team expertise.	Did not infer a uniform dose–response; summarised ground and air evidence narratively.
**Mortality**	Neonatal, early neonatal, transport-related, in-hospital, pre-discharge, or follow-up mortality.	Different risk windows alter event rates and prevent direct pooling.	Reported the study-defined endpoint and avoided combining non-equivalent mortality windows.
**Bronchopulmonary dysplasia**	Oxygen requirement at 28 days, at 36 weeks' postmenstrual age, or newer severity-based definitions.	Diagnostic thresholds and survival to assessment affect comparability.	Interpreted BPD directionally and within gestational-age context.
**Necrotising enterocolitis**	Any NEC versus Bell stage II or higher; clinical versus surgically confirmed disease.	Broader definitions produce higher event rates and may mix outcomes of different severity.	Preferred severe/stage-defined outcomes when available; no pooled estimate was calculated.
**Sepsis**	Culture-proven sepsis, clinically suspected sepsis, early-onset, late-onset, or combined infection outcomes.	Case definitions and surveillance periods vary, limiting direct comparison.	Maintained each study's definition and interpreted infection findings cautiously.
**Hypothermia**	Arrival temperature below 36.0 °C or 36.5 °C, any temperature below target, or severe hypothermia; different measurement sites and times.	Threshold and ascertainment differences influence prevalence and apparent intervention effects.	Focused on direction of effect and protocol context; did not combine heterogeneous thresholds.
**Transport-related adverse events**	Composite events, unintended clinical events, equipment incidents, airway events, desaturation, or physiological deterioration.	Event definitions, active surveillance, and reporting intensity differ between services.	Compared like-for-like outcomes where possible and treated service evaluations as context-dependent.
**Neurodevelopmental impairment**	Different ages at assessment, instruments, domains, severity thresholds, and composite definitions with death.	Follow-up duration, attrition, and measurement differences reduce comparability.	Synthesised long-term outcomes separately and emphasised adjustment for gestational age and antenatal factors.
**Analytical approach and risk of bias**	Unadjusted comparisons, multivariable models, matching/propensity methods, and variable handling of missing data.	Residual confounding and selection bias can explain part of the observed association.	Weighted conclusions qualitatively by design, sample size, adjustment, risk of bias, consistency, and directness.

Abbreviations: AAP, American Academy of Pediatrics; BPD, bronchopulmonary dysplasia; ELBW, extremely low birth weight; NEC, necrotising enterocolitis; SWiM, Synthesis Without Meta-analysis; VLBW, very low birth weight.
